# Risk factors for cement leakage after percutaneous vertebral augmentation for osteoporotic vertebral compression fractures: a meta-analysis

**DOI:** 10.1097/JS9.0000000000001895

**Published:** 2024-07-08

**Authors:** Yu Wu, Zelin Zhou, Guoliang Lu, Linqiang Ye, Aotian Lao, Shuai Ouyang, Zefeng Song, Zhigang Zhang

**Affiliations:** a Department of Orthopaedics, Dongguan Hospital of Traditional Chinese Medicine, Dongguan; b The First Clinical Medical School of Guangzhou University of Chinese Medicine, Guangzhou; c Medical Department, Dalian University of Technology, Dalian, P.R. China

**Keywords:** cement leakage, meta-analysis, risk factors, osteoporotic vertebral compression fractures, percutaneous vertebral augmentation

## Abstract

**Background:**

Osteoporotic vertebral compression fractures (OVCF) may necessitate percutaneous vertebral augmentation (PVA), a procedure not without its risks. One notable complication is cement leakage (CL), which can cause significant distress in patients. Despite its clinical importance, there remains a paucity of meta-analyses investigating these complications and their management in the existing literature.

**Material and methods:**

The authors systematically reviewed PubMed, Cochrane Library, Embase, and Web of Science databases up to February 2024 to identify studies examining CL following PVA treatment in OVCF. The authors assessed the quality of eligible cohort studies using the Newcastle–Ottawa Scale (NOS), extracted data on incidence, identified risk factors for CL, and conducting meta-analysis with Revman 5.2 software. The authors calculated odd ratios (OR) and mean differences (MD) with 95% CI applying random-effects models.

**Results:**

The authors identified twelve cohort studies that matched our strict inclusion criteria. These studies included a total of 2388 patients and 3392 vertebrae. CL was identified in 1132 vertebrae. Notable risk factors for CL included compromised cortical bone integrity (OR 5.00, 95% CI 3.01–8.29, *P*<0.00001), presence of intravertebral vacuum clefts (OR 1.68, 95% CI 1.07–2.65, *P*=0.03), basivertebral foramen sign (OR 1.77, 95% CI 1.09–2.89, *P*=0.02), and volume of cement used (MD 0.75, 95% CI 0.41–1.10, *P*<0.0001).

**Conclusion:**

The authors’ findings underscore the significance of cortical bone integrity, intravertebral vacuum cleft, basivertebral foramen sign, and cement volume as principal determinants of CL risk in PVA for OVCF. These insights advocate for tailored surgical strategies to mitigate the risk of CL in this patient population.

## Introduction

Highlights

- This is the first meta-analysis to explore the risk factors for cement leakage after percutaneous vertebral augmentation for osteoporotic vertebral compression fractures.
- The meta-analysis identified compromised cortical bone integrity, the presence of intravertebral vacuum clefts, and basivertebral foramen signs as significant anatomical risk factors for cement leakage.
- Distinct from prior meta-analyses, this review lies in its focused analysis on patients with osteoporotic vertebral compression fracture (OVCF), excluding those with spinal tumors, infections, or deformities, which can enhance the specificity of our findings.

Osteoporosis is considered one of the top 10 most significant worldwide health risks, according to the WHO^1,2^. The prevalence of osteoporotic vertebral compression fractures (OVCF) is escalating in the context of worldwide population aging, affecting over 1.4 million individuals globally each year^3,4^. These fractures contribute significantly to morbidity among the elderly, leading to lower back pain, reduced mobility, spinal deformity, and secondary complications including gastrointestinal and pulmonary dysfunction, clinical depression, and lower extremity weakness^1,5,6^.

Percutaneous vertebral augmentation (PVA), encompassing both percutaneous vertebroplasty (PVP) and percutaneous kyphoplasty (PKP), has emerged as the predominant minimally invasive intervention for OVCF, showing promising clinical outcomes^7,8^. Nonetheless, akin to other invasive techniques, PVA carries inherent risks, with cement leakage being a frequent complication. Data reveal leakage rates for PVP range between 56 and 87.5%, while PKP exhibits rates of 20–34%^9,10^. Such leakage, particularly into the intervertebral disc, heightens the risk of adjacent vertebral fractures^11,12^. Moreover, cement extravasation into the epidural or intervertebral foramen may compress spinal and nerve roots, elevating the risk of adjacent vertebral body fractures, nerve root compression symptoms, and in severe cases, paraplegia^13–15^. Furthermore, paraspinal vein cement extravasation poses risks for severe complications, including pulmonary cement embolism (PCE), cardiac perforation, cerebral embolism, and even fatality. In the previous literature, the incidence of PCE ranged from 0.3 to 23%^16–18^. Although there are no relevant epidemiological studies and specific clinical data on the incidence of other serious complications, clinical cases have been reported constantly^19–22^.

Considering these factors, it is crucial for doctors to give priority to preventive measures against cement leakage (CL) before and after percutaneous vertebral augmentation (PVA). The insights derived from this study aim to minimize preoperative CL and to predict intraoperative CL, thereby broadening the understanding of CL prevention following PVA. Such knowledge is crucial for informing the development and refinement of predictive tools for CL.

## Materials and methods

### Protocol and registration

The protocol was prospectively registered on PROSPERO (CRD42023472069). We are reporting our findings according to the updated guidance for the PRISMA guidance^23^. The detailed PRISMA checklist is provided for reference as Supplemental digital content A, Supplemental Digital Content 1, http://links.lww.com/JS9/D54.

### Study search strategy

We searched PubMed, Embase, Cochrane Library, and Web of Science for relevant studies published up to 1 February 2024. To collect studies on the incidence and risk factors of cement leakage after augmentation for osteoporotic vertebral compression fractures, we used the following search terms in the title, abstract or keyword: “Osteoporotic vertebral compression fractures”, “OVCF”, “OVCRF” OR “Osteoporotic spinal fractures” AND “percutaneous vertebroplasty”, “PVP” or “percutaneous kyphoplasty”, “PKP” or “Percutaneous vertebral augmentation”, “PVA” AND “cement leakage” OR “cement leakage”. The search strategy was adjusted according to the system characteristics of different databases. For the complete search, see supplemental digital content B, Supplemental Digital Content 2, http://links.lww.com/JS9/D55. The search limits were English language, studies conducted in humans, and full text available.

### Selection criteria

We conducted a thorough examination of the citations in all the articles we found to manually identify any additional articles that were relevant to our study. Every detected article was methodically evaluated based on the predetermined criteria for inclusion and exclusion. Studies were included if they met the following criteria: (1) study participants were OVCF patients treated with PVA; (2) studies were randomized controlled or observational studies, including cohort studies and case-control studies; (3) the study provided the incidence of CL and evaluate the risk factors related to CL (the number of risk factors analyzed was ≥2); (4) more than 50 participants were included in the study. We excluded case report, review article, technical report, non-English studies, study with ambiguous or unextractable data, and study of non-OVCF participants (e.g. spinal metastases, hemangioma, infections, or deformities). If there may be duplicate study populations in two articles, only the one with the most information was retained to avoid duplication of information.

### Data extraction

During our systematic review, two independent reviewers carefully collected data from each trial, paying close attention to a wide range of characteristics that are essential for our analysis. The foundational data included the first author’s name, publication year, study’s country of origin, and study design, which established the context and methodological framework of the research. We paid particular attention to risk factors consistently identified across studies, categorizing them into general information such as patient demographics characteristics (age, gender), BMI, bone mineral density (BMD), and medical history including diabetes, hypertension, and prior anti-osteoporosis treatments. Details regarding surgical history, specifically pedicle screw fixation, steroid medication, fracture segment, timeliness of the fracture, and the intervals from hospitalization to surgery as well as from injury to surgery, were also compiled. Preoperative imaging-related variables were assessed, including the height of the compressed vertebral body, fracture severity, cortical bone integrity, presence of intravertebral vacuum clefts, basivertebral foramen sign, and Cobb angle measurements. Lastly, surgery-related variables such as the approach selection, surgical method, cement volume and viscosity, and operation time were meticulously recorded.

According to Genant *et al.* ^24^, fracture severity was categorized into mild, moderate, and severe based on the reductions of 20–25%, 26–40%, and greater than 40%, respectively. In this study, reductions of less than 20% were also classified as mild. The intravertebral cleft was defined an abnormal, well-demarcated, linear or cystic hypointensity like air on MRI T1-weighted sequences and/or hyperintensity similar to cerebrospinal fluid on MRI T2 short-tau inversion recovery sequences^25^. Cortical disruption was characterized by clear discontinuity observed at the endplates or vertebral body wall on preoperative MRI or computed tomography (CT) scans. The basivertebral foramen sign is characterized by the passage of the vertebrobasilar vein through the center of the vertebral body’s posterior wall, resulting in a triangular or irregular quadrilateral bone defect on CT sagittal images or a porous defect in the bone on CT axial images^14^. As for bone cement viscosity, two different types of PMMA bone cement were used, namely low-viscosity PMMA and medium-viscosity PMMA.

Using DR, CT, or MRI to assess the occurrence of bone cement leakage, which was characterized as the presence of any cement outside the vertebral body. The risk factors were dependent variables, and the leakage of bone cement was an independent variable.

### Quality assessment

The study obtained from each database was imported into EndNote software, and any duplicate study was removed. Two autonomous reviewers evaluated the study based on the specified criteria for inclusion and exclusion. They initially excluded the title and abstract and then conducted a thorough examination of the complete text of the study that potentially fulfilled the requirements. The studies that might meet the requirements were cross-compared by two reviewers independently (if there were any differences, the reviewers would discuss with the third reviewer to decide whether to include the studies). The Newcastle–Ottawa scale (NOS) was adopted to assess the methodological quality of the articles included^26^. A total score of 9 was used, with greater than or equal to 7 as high-quality studies, 6 as moderate-quality studies, and less than or equal to 5 as low-quality studies. In case of disagreement during the evaluation process, the third researcher participated in the consensus meeting and reached a consensus.

### Statistical analysis

All data analyses were performed using RevMan 5.2 (Nordic Cochrane Center, Copenhagen, Denmark). Risk difference (RD) was used for single rate, odd ratios (OR) for dichotomous variables, and mean difference (MD) for continuous variables, and 95% CI was calculated. Heterogeneity was calculated with *I* ^2^ statistics. Due to the small number of articles included, the values of heterogeneity were not considered, and the random-effects model was adopted. *P* values less than 0.05 were considered statistically significant. Subgroup analysis was further performed according to the surgical procedures (PVP, PKP, and PKP/PVP)

### Assessment of publication bias

The presence of publication bias was assessed using Begg’s funnel plot, where the possibility of publication bias was considered small when the funnel plot was symmetric, and large otherwise. Typically, the abscissa represents the effect size of an individual research, while the ordinate represents the sample size. If the sample size is small and the precision of the study is low, it is distributed at the bottom of the funnel plot and scattered around. If the sample size is large and the study precision is high, it will be distributed at the top of the funnel and concentrated in the middle.

## Results

### Literature search and study characteristics

**Figure 1:**
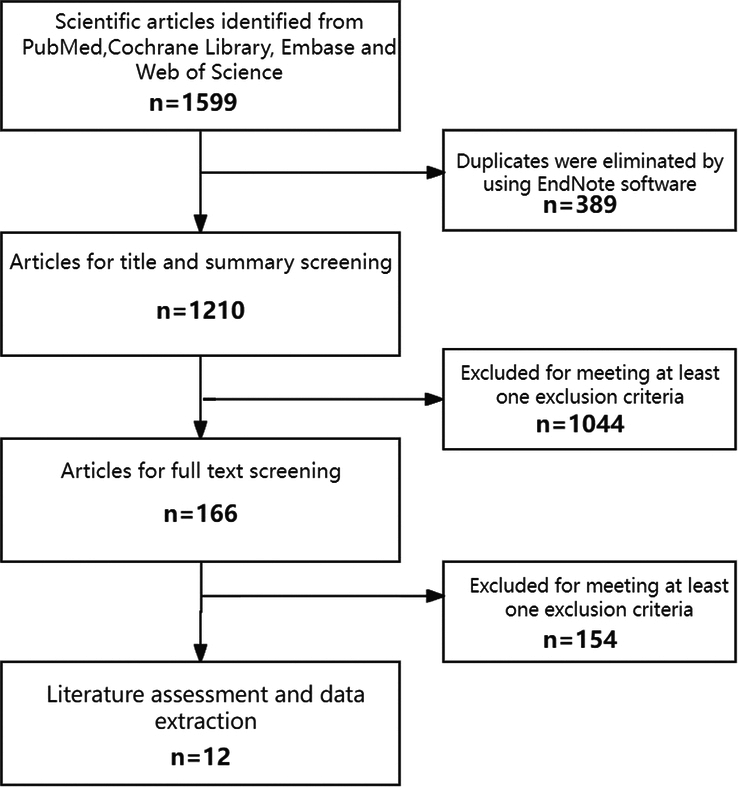
The flow diagram of literature selection.

A total of 1599 studies were initially retrieved according to the search strategy, and 389 duplicate studies were eliminated by EndNote software. A total of 1044 studies that did not meet the inclusion criteria were eliminated by reading the title and abstract, and 154 studies that did not meet the inclusion and exclusion criteria were further eliminated by carefully reading the full text. A total of 12 articles were included^27–38^, all of which were cohort studies. The study search process is shown in Figure 1, and the characteristics of the study included are shown in Table 1.

**Table 1:** Characteristics of included studies.

References	Country	Study design	Sample size (CL/non-CL)	Procedure	Investigated risk factors
Xie *et al.* ^27^	China	Retrospective	(100/182)	PVP/PKP	Age, sex, bone density, number of fractured vertebrae, number of treated vertebrae, fracture severity, operative approach, cement volume, preoperative vertebral compression ratio, preoperative local kyphosis angle, preoperative intraosseous clefts and preoperative vertebral cortical bone defect.
Ding *et al.* ^28^	China	Retrospective	(227/65)	PVP	Age, sex, spinal deformity index, fracture level, fracture Age, fracture type, severity grade, the presence of a vertebral cleft, cortical disruption, viscosity of bone cement and surgical approach.
Zhong *et al.* ^29^	China	Retrospective	(93/237)	PVP	Age, sex, CT values of the spine, fracture type, presence of pre-existing OVCF, number of treated vertebrae, the location, severity of the vertebral body fracture, the presence of Kummell’s disease, preoperative presence of endplate cortical defects, and cemented vertebral body fraction.
Ren *et al.* ^30^	China	Retrospective	(46/240)	PVP/PKP	Age, sex, fracture duration, BMD, dynamic fracture mobility, operative approach, segment of fracture, peripheral vertebrae wall damage, IVC, cement volume, preoperative vertebral height, and preoperative kyphotic angle.
Hong *et al.* ^31^	Korea	Retrospective	(54/180)	PVP	Age, sex, BMD, endplate cortical disruption, abnormal T2 hyperintensity in adjacent discs, Kümmell’s disease, linear body fracture with extension to endplate, level of treated vertebral body and cement volume.
Shen *et al.* ^32^	China	Retrospective	(17/154)	PKP	Preoperative vertebral body height, preoperative Cobb angle, cement volume, freshness of vertebral fracture, operative approach, vertebral body wall integrality, and the location of operative vertebrae.
Nieuwenhuijse *et al.* ^33^	The Netherlands	Retrospective	(130/33)	PVP	Age, sex, Spinal deformity index, Fracture level, Fracture Age, Fracture type, Fracture severity, Presence of cleft, Cortical disruption, Cement viscosity.
Li *et al.* ^34^	China	Retrospective	(81/304)	PVP	Age, sex, BMI, BMD, cement volume, injury to surgery, hospitalization to surgery, surgery time, multiple vertebral fractures and steroid use.
Hu *et al.* ^35^	China	Retrospective	(258/254)	PVP/PKP	Age, BMI, BMD, time interval from injury to admission, time interval from admission to surgery, history of OVCF, surgical history of PVP/PKP, history of chronic diseases use of steroid medication, use of anti-osteoporosis medication, surgical history of pedicle screw fixation, number of fractured vertebral bodies, number of surgically treated vertebral bodies, and location of fracture (thoracic, thoracolumbar (T11–L2), lumbar, type of operation (PVP or PKP), puncture approach (unipedicular or bipedicular, the volume of bone cement, length of surgery time, anesthesia technique, IVC, cortical disruption, fracture type and severity, basivertebral foramen sign, the intrusion of the posterior wall.
Mirovsky *et al.* ^36^	Israel	Retrospective	(27/39)	PVP	Age, sex, deformity, fractured vertebra, fracture type, fracture Age, presence of a cleft and bone cement volume.
Li *et al.* ^37^	China	Retrospective	(72/445)	PKP	Age, sex, BMI, BMD, history of anti-osteoporotic therapy, hypertension, diabetes mellitus, functional status, damage factors, fracture segments, IVC, Schmorl’s nodules sign, basivertebral foramen’s sign, cortical bone defect, the grade of vertebral body compression, height of fracture vertebra, Cobb angle, history of PKP, operation time, number of vertebral fractures, surgical approach, stage of bone cement injection, balloon pressure, balloon volume, cement volume and cement distribution pattern.
Huang *et al.* ^38^	China	Retrospective	(27/117)	PKP	Age, BMI, marital status, past medical history, place of residence, degree of education, causes, site of injured vertebrae, use of zoledronic acid, fracture acuteness, peripheral vertebrae wall damage, cement volume, TNF-α, IL-6, BMD, PINP, BGP, vertebral height ratios of injured vertebrae, Cobb angle, VAS score, and ODI score.

BMD, bone mineral density; CL, cement leakage; CT, computed tomography; IVC, intravertebral vacuum cleft; ODI, The Oswestry disability index; OVCF, osteoporotic vertebral compression fracture; PKP, percutaneous kyphoplasty; PVP, percutaneous vertebroplasty; VAS, visual analog scale.

### Quality assessment

According to the NOS evaluation, among the 12 articles, 4 articles^27,29,32,38^ scored 7 points and 8 articles^28,30,31,33–37^ scored 8 points (Table 2), all of which were of high quality.

**Table 2:** Quality assessment of included studies according to the Newcastle–Ottawa scale.

References	Selection	Comparability	Exposure	Total Score
Xie *et al.* ^27^	2	2	3	7
Ding *et al.* ^28^	3	2	3	8
Zhong *et al.* ^29^	2	2	3	7
Ren *et al.* ^30^	3	2	3	8
Hong *et al.* ^31^	3	2	3	8
Shen *et al.* ^32^	3	2	2	7
Nieuwenhuijse *et al.* ^33^	3	2	3	8
Li *et al.* ^34^	3	2	3	8
Hu *et al.* ^35^	3	3	2	8
Mirovsky *et al.* ^36^	3	2	3	8
Li *et al.* ^37^	3	2	3	8
Huang *et al.* ^38^	2	2	3	7

### Meta-analysis results

#### Incidence

**Figure 2:**
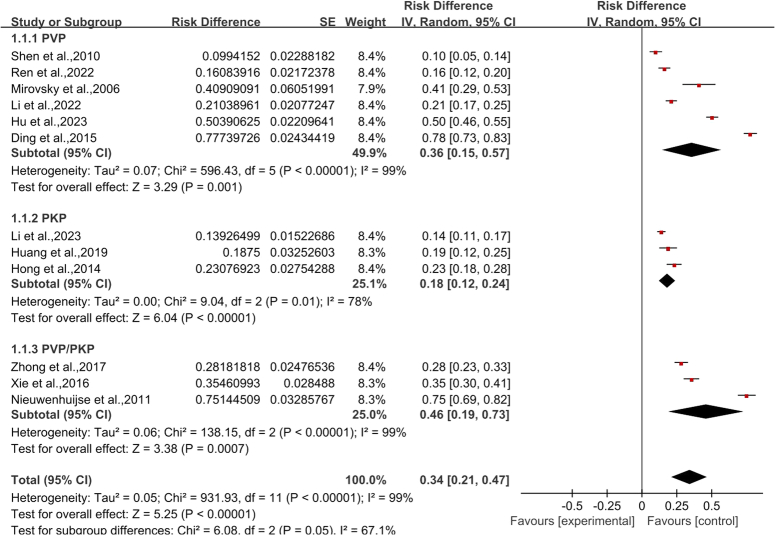
Incidence of cement leakage; IV, inverse variances (statistical method); PKP, percutaneous kyphoplasty; PVP, percutaneous vertebroplasty.

A total of 12 articles were included in this study, with a total of 3392 OVCF vertebral bodies, of which 1132 vertebral bodies developed CL after PVA. Heterogeneity test was performed (*P*<0.00001, *I* ^2^=99%), and sensitivity analysis was used to explore the source of heterogeneity. After excluding the included studies one by one, the heterogeneity of each group was still high, indicating that the results were reliable, and the random effect model was used. The overall incidence of CL was 34% (RD 0.34 95% CI 0.21–0.47; Fig. 2). Among them, the incidence of CL following PVP was 36% (95% CI 0.15–0.57) and PKP was 18% (95% CI 0.12–0.24).

#### Age

The study of age included a total of seven articles^28,30,34–38^, with a combined sample size of 2170 patients. Among them, there were 736 cases in the CL group and 1434 cases in the non-CL group. The heterogeneity test (*P*=0.005, *I* ^2^=67%) was performed. The results showed no significant difference between the two groups (MD −1.31, 95% CI −2.69 to 0.07, *P*=0.06; Table 3).

**Table 3:** Meta analysis of factors affecting cement leakage after percutaneous vertebral augmentation for osteoporotic vertebral compression fractures.

Items	Studies number (sample size)	Heterogeneity analysis		Meta-analysis result		
		*P*	*I* ^*2*^	OR/MD	95% CI	*P*
Age	7 (2170)	0.005	67%	MD −1.31	−2.69 to 0.07	0.06
Sex (male/female)	7 (2388)	1.00	0%	OR 0.94	0.75–1.19	0.63
BMI	3 (1041)	0.75	0%	MD 0.20	−0.23 to 0.63	0.37
BMD	3 (1183)	0.02	74%	MD −0.01	−0.18 to 0.17	0.95
History of diabetes	2 (629)	0.27	17%	OR 1.11	0.59–2.08	0.75
History of hypertension	2 (629)	0.17	48%	OR 1.29	0.62–2.69	0.50
History of anti-osteoporosis treatment	3 (1141)	0.89	0%	OR 0.87	0.55–1.36	0.53
History of pedicle screw internal fixation	2 (999)	0.99	0%	OR 0.91	0.53–1.57	0.73
History of steroid use	2 (897)	0.80	0%	OR 0.59	0.33–1.05	0.07
Fracture segment (TL/non-TL)	5 (1751)	0.29	19%	OR 1.12	0.86–1.47	0.40
Fracture duration (acute/subacute)	2 (283)	0.77	0%	OR 0.97	0.49–1.90	0.92
Hospitalization to surgery	2 (897)	0.18	44%	MD 0.17	−0.35 to 0.69	0.52
Injury to surgery	3 (1183)	0.89	0%	MD −0.19	−3.30 to 2.92	0.91
Compressed vertebral body height	3 (430)	0.07	63%	MD −2.29	−6.08 to 1.49	0.24
Fracture severity (mild/moderate or severe)	6 (1999)	0.01	66%	OR 0.76	0.51–1.12	0.16
Cortical bone integrity	9 (2617)	0.001	74%	OR 5.00	3.01–8.29	<0.00001
Intravertebral vacuum cleft	8 (2448)	0.0004	74%	OR 1.68	1.07–2.65	0.03
Basivertebral foramen sign	9 (1029)	0.20	39%	OR 1.77	1.09–2.89	0.02
Cobb angle	3 (832)	<0.0001	90%	MD 2.09	−0.89 to 5.07	0.17
Approach selection (unilateral/bilateral)	3 (1200)	=0.006	80%	OR 1.10	0.50–2.43	0.82
Surgical method (PVP/PKP)	3 (1180)	<0.0001	91%	OR 1.14	0.38–3.41	0.81
Cement volume	6 (1564)	0.005	70%	MD 0.75	0.41–1.10	<0.0001
Cement viscosity (low/middle)	2 (417)	0.14	53%	OR 1.74	0.78–3.89	0.18
Operation time	2 (897)	0.04	76%	MD 3.34	−3.43 to 10.11	0.33

BMD, bone mineral density; MD, mean difference; OR, odd ratio; PKP, percutaneous kyphoplasty; PVP, percutaneous vertebroplasty.

#### Sex

Seven articles^28–30,34–37^ were included in the analysis of gender, including 2388 patients, 599 cases in the male group and 1789 cases in the female group. Heterogeneity test was performed (*P*=1.00, *I* ^2^=0%), and the results showed no statistical difference between the two groups. (OR 0.94, 95% CI 0.75–1.19, *P*=0.63; Table 3).

#### BMI

Three articles^34,35,38^ of BMI were included in the analysis, with a total of 1041 patients, including 366 cases in CL group and 675 cases in non-CL group. Heterogeneity analysis was performed (*P*=0.75, *I* ^2^=0%), the results showed no statistical difference between the two groups. (MD 0.20, 95% CI −0.23 to 0.63, *P*=0.37; Table 3).

#### Bone mineral density

The analysis included three articles^30,34,35^ on BMD, which had a total of 1183 patients. Among these patients, there were 385 cases in the CL group and 798 cases in the non-CL group. Heterogeneity analysis was performed (*P*=0.02, *I* ^2^=74%). The results were not statistically different between the two groups. (MD −0.01, 95% CI −0.18 to 0.17, *P*=0.95; Table 3).

#### History of diabetes

Two articles^37,38^ were included in the analysis. A total of 629 patients were included in the analysis, including 97 cases in CL group and 532 cases in non-CL group. Heterogeneity analysis was performed (*P*=0.27, *I* ^2^=17%), and the results were not statistically significant between the two groups (OR 1.11, 95% CI 0.59–2.08, *P*=0.75; Table 3).

#### History of hypertension

The analysis included two papers^37,38^ with a total of 629 patients. Among them, there were 97 cases in the CL group and 532 instances in the non-CL group. Heterogeneity analysis was performed (*P*=0.17, *I* ^2^=48%), and the results were not statistically significant between the two groups (OR 1.29, 95% CI 0.62–2.69, *P*=0.50; Table 3).

#### History of anti-osteoporosis treatment

Three articles^35,37,38^ were included in the analysis, with a total of 1141 patients, including 355 patients in the CL group and 786 patients in the non-CL group. Heterogeneity analysis was performed (*P*=0.89, *I* ^2^=0%). The results were not statistically significant between the two groups (OR 0.87, 95% CI 0.55–1.36, *P*=0.53; Table 3).

#### History of pedicle screw internal fixation

The analysis included two articles^35,38^ with a total of 999 patients. Among them, there were 330 cases in the CL group and 699 instances in the non-CL group. Heterogeneity analysis was performed (*P*=0.99, *I* ^2^=0%). The results were not statistically significant between the two groups (OR 0.91, 95% CI 0.53–1.57, *P*=0.73; Table 3).

#### Steroid medication

Two articles^34,35^ were included in the analysis, with a total of 897 patients, including 339 patients in the CL group and 558 patients in the non-CL group. Heterogeneity analysis (*P*=0.80, *I* ^2^=0%) showed no heterogeneity. The results were not statistically significant between the two groups (OR 0.59, 95% CI 0.33–1.05, *P*=0.07; Table 3).

#### Fracture segment

Five articles^28,30,35,37,38^ on fracture segment were included in the analysis, with a total of 1751 vertebral bodies, including 1099 cases in the TL group and 652 cases in the non-TL group. Heterogeneity analysis was performed (*P*=0.29, *I* ^2^=19%). The results were not statistically significant between the two groups (OR 1.12, 95% CI 0.86–1.47, *P*=0.40; Table 3).

#### Fracture duration

The analysis included two articles^32,38^ comprising a total of 283 patients. Among these, there were 152 instances in the acute group and 131 cases in the subacute group. Heterogeneity analysis was performed (*P*=0.77, *I* ^2^=0%). The results were not statistically significant between the two groups (OR 0.97, 95% CI 0.49–1.90, *P*=0.92; Table 3).

#### Hospitalization to surgery

The analysis included two articles^34,35^ with a total of 897 patients. Among them, there were 339 instances in the CL group and 558 cases in the non-CL group. Heterogeneity analysis was performed (*P*=0.18, *I* ^2^=44%) and the results were not statistically significant between the two groups (MD 0.17, 95% CI −0.35 to 0.69, *P*=0.52; Table 3).

#### Injury to surgery

The analysis included three articles^30,34,35^, which had a total of 1183 patients. Among these patients, there were 385 cases in the CL group and 798 cases in the non-CL group. Heterogeneity analysis was performed (*P*=0.89, *I* ^2^=0%), there was no heterogeneity between the two groups. The results were not statistically significant between the two groups (MD −0.19, 95% CI −3.30 to 2.92, *P*=0.91; Table 3).

#### Compressed vertebral body height

The research included three articles^30,32,38^ that together examined 430 vertebral bodies. Of these, 73 instances were in the CL group and 357 cases were in the non-CL group. Heterogeneity analysis showed high heterogeneity (*P*=0.07, *I* ^2^=63%). The results showed no significant difference between the two groups (MD −2.29, 95% CI −6.08 to 1.49, *P*=0.24; Table 3).

#### Fracture severity

Six articles^27–29,35–37^ on fracture severity were included in the analysis, with a total of 1999 vertebral bodies, including 928 cases in the Mild group and 1071 cases in the Moderate/Severe group. Heterogeneity analysis was performed (*P*=0.01, *I* ^2^=66%) and no statistical difference was found between the two groups (OR 0.76, 95% CI 0.51–1.12, *P*=0.16; Table 3).

#### Cortical bone integrity

**Figure 3:**
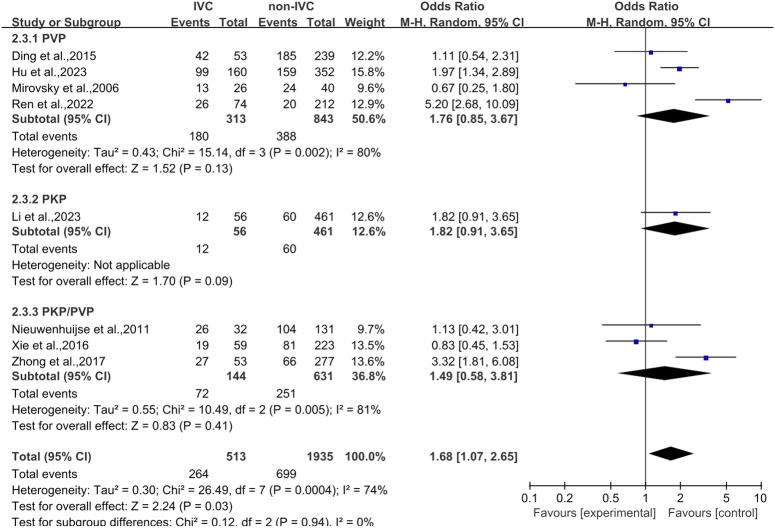
Forest plot of cortical integrity. IVC, intravertebral vacuum cleft; PKP, percutaneous kyphoplasty; PVP, percutaneous vertebroplasty.

The analysis comprised nine articles^28–33,35,37,38^ that examined cortical bone integrity. A total of 2617 vertebral bodies were included, with 938 instances in the incomplete cortex group and 1679 cases in the intact cortex group. Heterogeneity analysis was performed (*P*=0.0001, *I* ^2^=74%). The risk of cement leakage after PVA was five times higher in vertebral bodies with cortical destruction than in vertebral bodies without cortical destruction. (OR 5.00, 95% CI 3.01–8.29, *P*<0.00001; Fig. 3).

#### Intravertebral vacuum cleft

**Figure 4:**
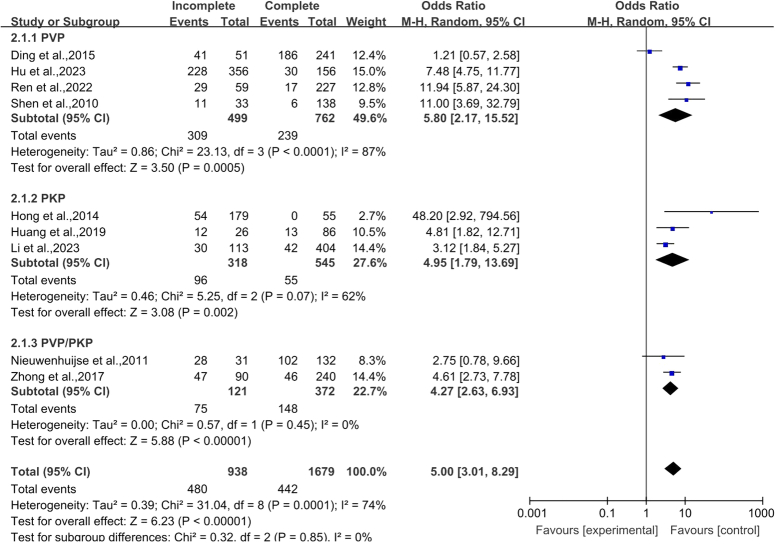
Forest plot of intravertebral vacuum cleft. PKP, percutaneous kyphoplasty; PVP, percutaneous vertebroplasty.

There were a total of 2448 vertebral bodies analyzed in eight articles^27–30,33,35–37^. Among these, 513 instances were in the IVC group and 1935 cases were in the non-IVC group. Heterogeneity analysis was performed (*P*=0.0004, *I* ^2^=74%). The incidence of CL in the vertebral body with IVC was more than twice as high as that in the vertebral body without IVC. (OR 1.68, 95% CI 1.07–2.65, *P*=0.03; Fig. 4).

#### Basivertebral foramen sign

The analysis included two articles^35,37^, which had a total of 1029 vertebral bodies. Among these, there were 330 instances in the CL group and 699 cases in the non-CL group. Heterogeneity analysis was performed (*P*=0.20, *I* ^2^=39%). The result suggested that the risk of CL in the vertebral body with the basivertebral foramen sign was more than 1.8 times higher than that in the vertebral body without the basivertebral foramen sign. (OR 1.77, 95% CI 1.09–2.89, *P*=0.02; Table 3).

#### Cobb angle

The study included three studies^32,37,38^, which involved a total of 832 vertebral bodies. Among them, there were 116 cases in the CL group and 716 cases in the non-CL group. Heterogeneity analysis was performed (*P*<0.0001, *I* ^2^=90%). The result indicates that the Cobb angle was not a risk factor for CL. (MD 2.09, 95% CI −0.89 to 5.07, *P*=0.17; Table 3).

#### Approach selection

The analysis included three articles^32,35,37^, which collectively examined a total of 1200 vertebral bodies. Among them, there were 357 cases in the unilateral group and 843 cases in the bilateral group. Heterogeneity analysis was performed (*P*=0.006, *I* ^2^=80%). There was no significant difference between the two groups. (OR 1.10, 95% CI 0.50–2.43, *P*=0.82; Table 3).

#### Surgical method

The study included three studies^27,30,35^, which involved a total of 1180 vertebral bodies. Among these, there were 443 instances in the PVP group and 737 cases in the PKP group. Heterogeneity analysis was performed (*P*<0.0001, *I* ^2^=91%). The result showed no significant difference between the two groups. (OR 1.14, 95% CI 0.38–3.41, *P*=0.81; Table 3).

#### Cement volume

**Figure 5:**
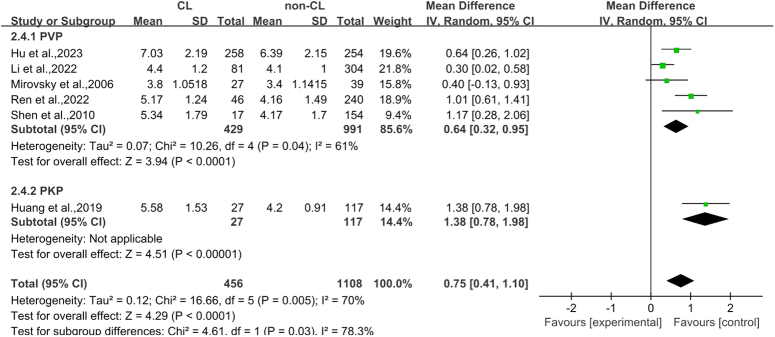
Forest plot of basivertebral foramen sign. CL, cement leakage; PKP, percutaneous kyphoplasty; PVP, percutaneous vertebroplasty.

The analysis included six articles^30,32,34–36,38^ that together examined 1564 vertebral bodies. Among them, there were 456 cases in the CL group and 1108 cases in the non-CL group. Heterogeneity analysis was performed (*P*=0.005, *I* ^2^=70%). The result showed that there was a statistical difference between the two groups (MD 0.75, 95% CI 0.41–1.10, *P*<0.0001; Fig. 5).

#### Cement viscosity

Two articles^28,33^ on cement viscosity were included in the analysis, with a total of 417 vertebral bodies, including 159 cases in the low bone cement density group and 258 cases in the middle bone cement density group. Heterogeneity analysis (*P*=0.14, *I* ^2^=53%) showed that the heterogeneity was high, and the results were not statistically significant between the two groups (OR 1.74, 95% CI 0.78–3.89, *P*=0.18; Table 3).

#### Operation time

The study included just two studies^34,35^, which had a total of 897 vertebral bodies. Among them, there were 339 instances in the CL group and 558 cases in the non-CL group. Heterogeneity analysis was performed (*P*=0.04, *I* ^2^=76%). The result showed that no statistical difference was found between the two groups (MD 3.34, 95% CI −3.43 to 10.11, *P*=0.33; Table 3).

### Subgroup analysis

According to the results of this study and previous studies^9,10^, it is suggested that the incidence of CL in OVCF is not the same with different surgical methods (PVP and PKP). Therefore, we conducted subgroup analysis of the risk factors derived from the meta-analysis based on different surgical methods and excluded the deviation of results caused by differences in surgical methods. Since only two studies on basivertebral foramen sign were included, no subgroup analysis was performed. The results showed that cortical bone integrity and cement volume had statistical significance in different operation groups (Fig. 3, Fig. 5), while intravertebral vacuum cleft had no statistical significance in either operation groups (Fig. 4).

### Publication bias

**Figure 6:**
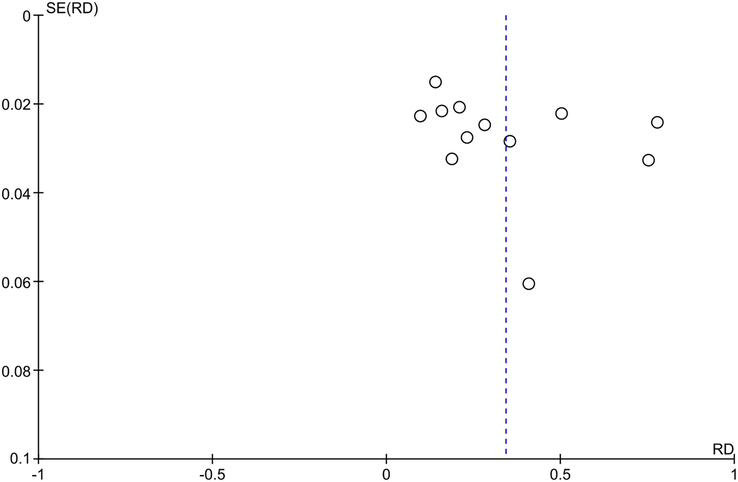
Publication bias based on incidence rate. RD, risk difference.

We rigorously assessed publication bias concerning the incidence of CL. The funnel plot depicted in Figure 6, characterized by its symmetry, suggests a minimal risk of publication bias among the studies considered. This symmetry implies that the findings are likely representative of the true effects despite the potential for heterogeneity arising from variances across different medical centers, as well as discrepancies in surgical indications and techniques. This assessment underscores our confidence in the robustness of our data, albeit acknowledging the inherent diversity in clinical practice settings.

## Discussion

PVA is renowned for its minimally invasive methodology, which has been scientifically demonstrated to swiftly and efficiently relieve the pain linked to OVCF^7,8,14^. Nonetheless, the procedure is not devoid of complications, with CL emerging as the most prevalent postoperative issue. The underlying mechanism of CL involves the injection of bone cement in a liquid state, which naturally seeks paths of least resistance through the bone channels. During its polymerization process, the cement heats releases toxic monomers, and solidifies into a non-degradable mass^39^. Unintended dispersion into non-target areas can severely damage adjacent tissues, precipitating a cascade of complications. Documented cases highlight the grave outcomes of CL, including peripheral nerve compression, spinal cord injuries, pulmonary embolism, and cardiac embolism^16–18^, with intervertebral CL notably elevating the risk of fractures in adjacent vertebrae^11,12^. It is imperative for spinal surgeons to remain vigilant of the CL risk associated with PVA to mitigate the incidence of these adverse events. In our analysis, the observed incidence of CL following PVA in OVCF patients was 34% (95% CI 21–47%), aligning with previously reported rates^15,40,41^. Among them, the incidence of CL following PVP was 36% (95% CI 0.15–0.57) and PKP was 18% (95% CI 0.12–0.24).

While various classification schemes for CL exist, no universal standard has been adopted. The anatomical location-based classification remains a commonly utilized standard in clinical settings. In this analysis, we employed the classification method proposed by Yeom *et al.* ^42^, which categorizes CL based on the route of leakage into type B (vertebrobasilar vein), type S (transvertebral segmental vein), and type C (cortical bone), with intervertebral disc leakage designated as a specific subtype of type C^43^. The distribution of leakage types in our study was 17.04% (type B), 24.81% (type S), and 58.15% (type C), findings that are in concordance with those from previous investigations^44–46^.

### Demographics characteristics

In our meta-analysis, no independent risk factors related to demographic characteristics for CL following PVA in patients with OVCF were identified, aligning with the outcomes of the studies reviewed. Nevertheless, one study within our analysis identified high BMD as a potential risk factor for CL^27^. This study posited that vertebra with higher BMD resists cement injection more strongly, necessitating increased injection pressure. In addition, they contended that a higher BMD is associated with a more compact network of bone structures and narrower gaps between these structures. This restricts the amount of bone cement that can be inserted and requires more space for the same volume of cement, thereby increasing the likelihood of leakage into the surrounding bone structures. Conversely, another study we included argued that low BMD, indicative of severe osteoporosis, could increase the likelihood of CL due to the ease with which bone cement penetrates the vertebral venous system, spreading into the posterior vertebral sinus and segmental veins^37^. However, our meta-analysis concluded that BMD does not constitute a significant risk factor for CL, highlighting the complexity of factors influencing CL risk and the need for further research to elucidate these relationships.

### Radiology-related variables

This meta-analysis corroborates previous findings that cortical bone integrity is a paramount predictor of CL in patients undergoing PVA for OVCF, as evidenced by numerous studies^28,33,35,37,38^. The fluid nature of bone cement predisposes it to leakage through fractures in an incomplete vertebral cortex, flowing along the spinal canal and exiting through compromised cortical areas, thereby elevating the risk of CL^28,29,47^.

Furthermore, the presence of IVC has been consistently identified as a significant risk factor for postoperative CL^29–31,33,35^. Originally linked with Kummell’s disease, IVC signifies vascular necrosis and severe vertebral collapse, indicative of fracture nonunion^45,48^. The presence of IVC facilitates the injection of bone cement into the intervertebral fissure due to reduced resistance, increasing the likelihood of CL^49^. This was further affirmed by Yu *et al.* ^50^, who observed a higher incidence of CL in patients with IVC compared to those without.

Additionally, our analysis identified the basivertebral foramen sign as a significant contributor to the risk of CL. This indication is identified by the vertebrobasilar vein passing through the posterior wall of the vertebral body. It is seen as a bone defect on CT imaging and can allow bone cement to leak, which may result in pulmonary embolism^14,18^. The anatomical structure of the basivertebral foramen, which is connected to many venous channels within the vertebral body, is responsible for both B-type and S-type cement leakage mechanisms^42,51,52^. Baumann *et al.* ^53^ proposed that when inserting a needle, it is important to consider the orientation of the vertebral body in order to avoid penetrating the endplate and reduce the risk of complications related to the cervical spine. The integrity of the vertebral cortex, the presence of IVC, and the basivertebral foramen sign thus create potential pathways for CL, allowing injected bone cement to escape the vertebral body. Moreover, Ren *et al.* ^30^ highlighted dynamic fracture mobility as a key factor influencing CL risk, a concept denoting the deformity’s automatic reduction in the prone position and indicating complete cortical and cancellous bone disruption^54–56^. McKiernan *et al.* ^55^ noted that prone hyperextension positioning could exacerbate perivertebral wall damage, thus increasing CL risk. Given the scarcity of studies on this aspect, further experimental and clinical research is warranted to deepen our understanding and develop more effective prevention strategies.

### Operation-related variables

Notably, in this comprehensive meta-analysis, the surgical approach was not a risk factor for CL after PVA in OVCF patients. Xie *et al.* ^27^ observed that the incidence of CL was 32.54% in PVP group and 43.84% in PKP group, while Hu *et al.* ^35^ reported that the incidence of CL in PVP group and PKP group was 41.03% and 52.07%, suggesting that the incidence of CL in PKP surgery had a higher trend, but did not reach statistical significance. Ren *et al.* ^30^ reported that the CL rate was 20.51% in PVP group and 10.77% in PKP group, and the difference was statistically significant (*P*=0.026). Meta-analysis of the three studies on surgical methods suggested that the results were very heterogeneous (*I* ^2^=91%), which could not be used as a reference for the superiority and inferiority of surgical methods. However, according to the results of this study, the incidence of CL following PVP was 36% (95% CI 0.15–0.57) and PKP was 18% (95% CI 0.12–0.24). There are indeed differences in the incidence of CL in OVCF treated by different surgical methods. Therefore, we conducted a subgroup analysis on the risk factors derived from the meta-analysis based on different surgical methods to exclude the deviation of results caused by differences in surgical methods. The results showed that the four risk factors obtained in this study still have guiding significance for the two different surgical methods.

Regarding cement volume, our findings corroborate previous studies indicating that larger cement volumes are associated with more pronounced pain relief but also with an increased risk of CL^57^. This association is attributed to the heightened intravertebral pressure resulting from larger cement volumes, promoting cement migration towards areas of lower pressure, and increasing CL risk^58^. Our analysis suggests a nuanced approach to cement volume determination, emphasizing the importance of tailored cement volumes based on the specific characteristics of the vertebral fracture and intraoperative observations, rather than a blanket increase in volume to maximize vertebral height restoration^59,60^.

In addition, the viscosity of the bone cement emerged as a critical topic in the study of cement leakage. While lower-viscosity cement may facilitate deeper trabecular penetration, it also raises the risk of extravasation^33^. Conversely, higher-viscosity cement tends to remain more localized within the trabeculae, reducing the likelihood of leakage^27^. Despite these insights, our analysis did not establish a significant correlation between cement viscosity and CL rates. Li *et al.* ^37^, posited that the timing of cement injection—specifically, opting for later stages when the cement exhibits a ‘stringy’ consistency—can markedly diminish CL risk. This observation underscores the importance of precise timing in the cementing process to mitigate CL risk. Furthermore, the correlation between filling pattern uniformity and cement viscosity suggests the complexity of evaluating the direct impact of viscosity on CL rates, highlighting the need for further investigation into this aspect^61^.

### Strengths and limitations

The merit of this review lies in its focused analysis on patients with OVCF, distinct from prior investigations^60^. By exclusively considering OVCF patients and excluding those with spinal tumors, infections, or deformities, we minimized potential confounding variables, enhancing the specificity of our findings. Furthermore, this study ventured beyond the scope of earlier meta-analyses^60^ by examining an expanded array of potential risk factors for CL. Notably, it is the first meta-analysis to identify the sign of basivertebral foramen as a significant risk factor for CL in OVCF patients undergoing PVA.

However, this review is not without its limitations. Dynamic fracture mobility^30^, high signal intensity on T2-weighted MRI of adjacent intervertebral discs^31^, and other factors were mentioned in the studies reviewed, but a comprehensive analysis was hindered by the scarcity of data. Additionally, the variability in surgical indications and techniques across different care centers introduced clinical heterogeneity, which necessitates a cautious interpretation of our findings. The lack of sufficient data and the impracticality of performing regression analyses limit our ability to draw definitive conclusions regarding the interplay among risk factors. Therefore, we advocate for the initiation of additional high-quality clinical studies to enrich the clinical data pool.

## Conclusion

Our meta-analysis revealed that insufficient cortical bone integrity, the existence of intravertebral vacuum clefts, the basivertebral foramen sign, and the volume of injected cement are crucial factors that significantly affect CL after PVA for OVCF. To mitigate the risk of CL, we recommend meticulous preoperative evaluation of imaging data and cautious cement injection under rigorous fluoroscopic guidance. Special attention is warranted for vertebrae exhibiting cortical incompleteness, intravertebral vacuum clefts, and basivertebral foramen signs as these features facilitate cement escape. Additionally, proper management of cement injection—both in timing and volume—is essential. The procedure should be halted upon cement approaching the vertebral body’s posterior edge or if leakage is observed, emphasizing that the quantity of cement injected should be judiciously controlled to balance efficacy with safety.

## Ethical approval

Our research does not require ethical review.

## Consent

Not applicable.

## Source of funding

The research was supported by Dongguan Science and Technology of Social Development Program (NO. 20231800936872).

## Author contribution

All authors contributed to the study conception and design. W.Y. performed literature search, W.Y. and Z.Z.L. reviewed articles for inclusion/exclusion with third-party arbitration by L.G.L. if required. Data collection was performed by O.Y.S. and L.A.T. and data analysis was performed by Z.Z.G., S.Z.F., and Y.L.Q. The first draft of the manuscript was written by W.Y. and all authors commented on previous versions of the manuscript. All authors read and approved the final manuscript.

## Conflicts of interest disclosure

The authors declare no conflict of interest.

## Research registration unique identifying number (UIN)

This meta-analysis was registered with the international prospective register of systematic reviews (PROSPERO) on 29 January 2024 (registration number CRD 42023472069).

## Guarantor

Not applicable.

## Data availability statement

All data generated or analyzed during this study are included in this published article (and its supplementary information files).

## Provenance and peer review

Not commissioned, externally peer-reviewed.

## Supplementary Material
