# Supplementary material for: Risk factors for cement leakage after percutaneous vertebral augmentation for osteoporotic vertebral compression fractures: a meta-analysis
Source: Int J Surg. 2024 Jul 8;111(1):1231–43. doi: 10.1097/JS9.0000000000001895 (PMC11745741; doi:10.1097/JS9.0000000000001895)

Age:


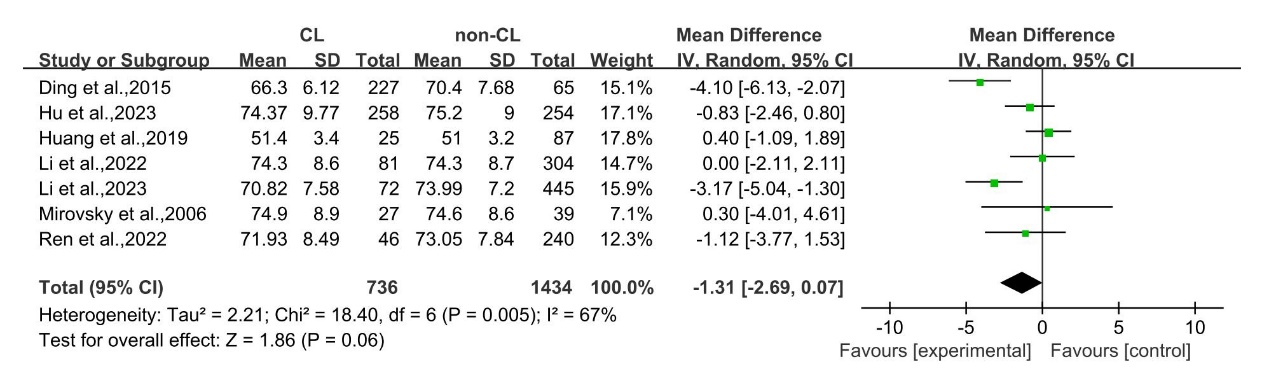


Approach selection:


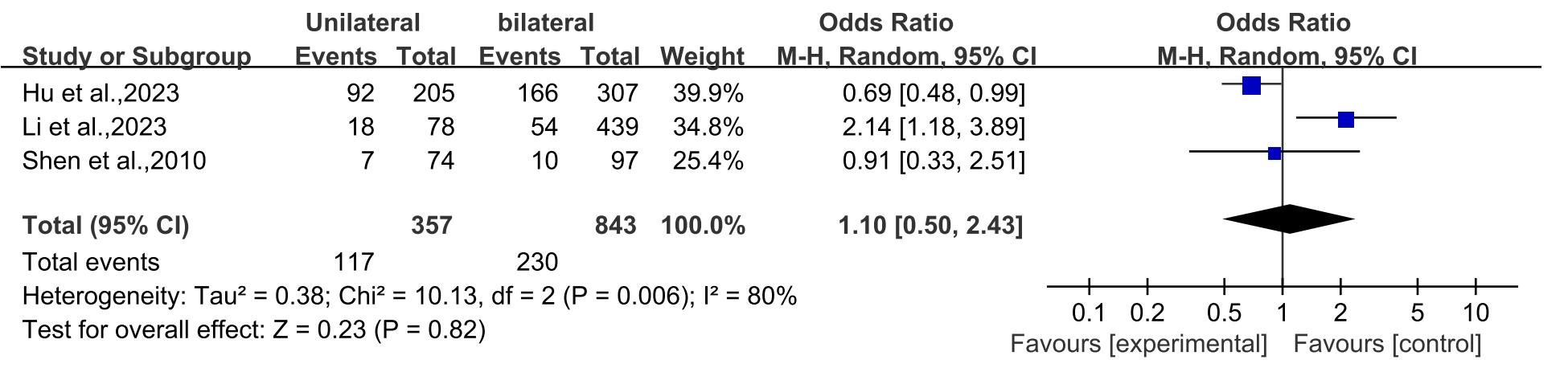


Basivertebral foramen sign:


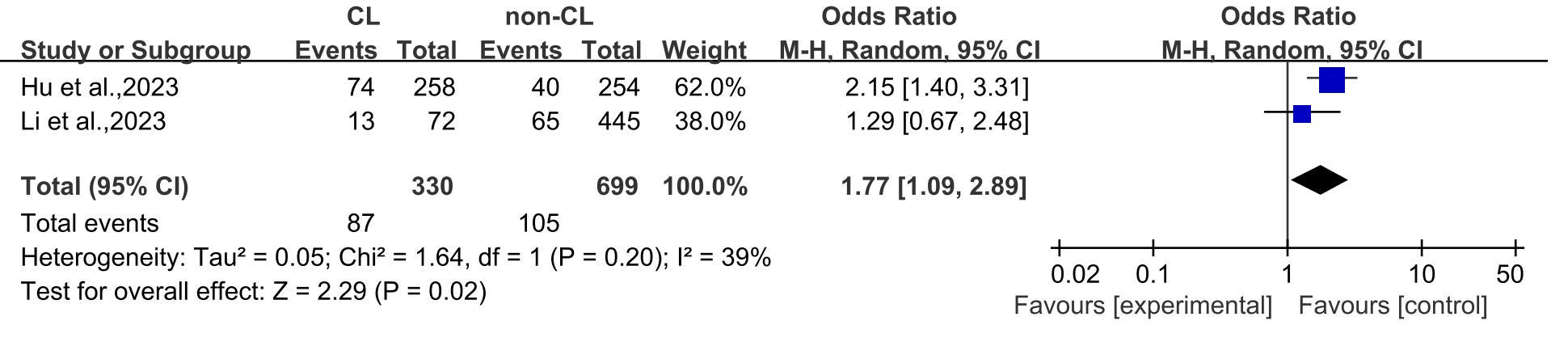


BMD:


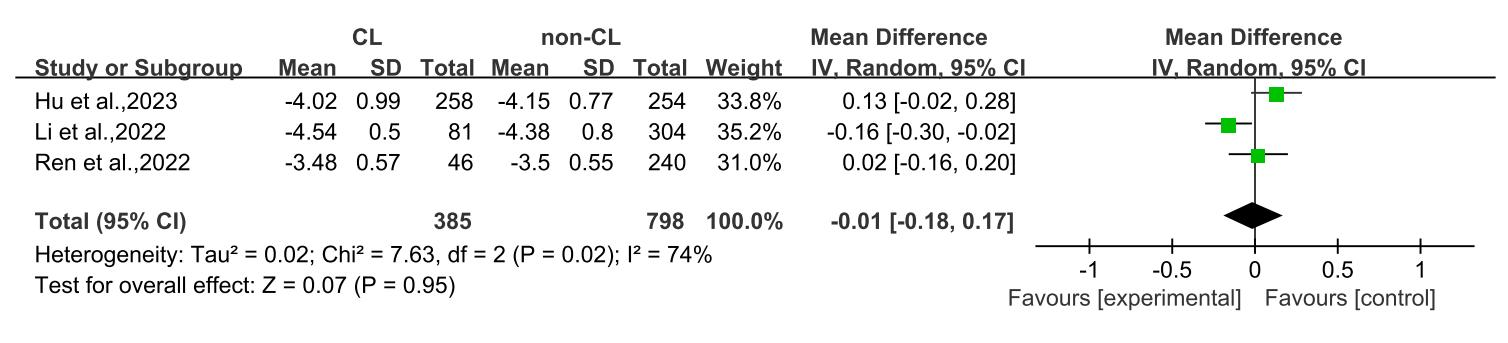


BMI:


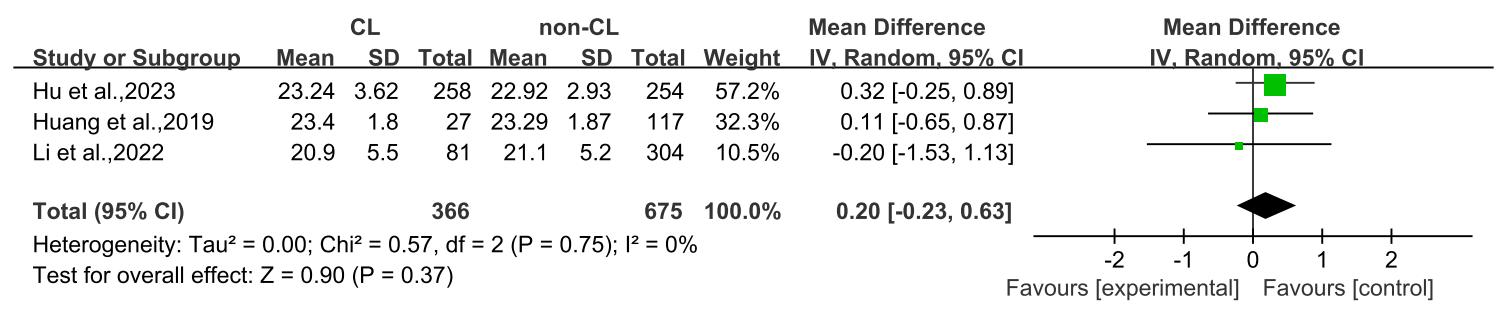


Cement viscosity:


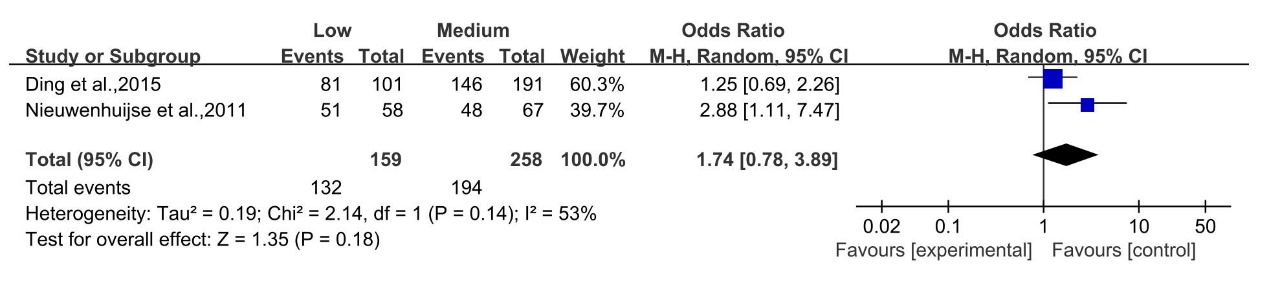


Cobb angle:


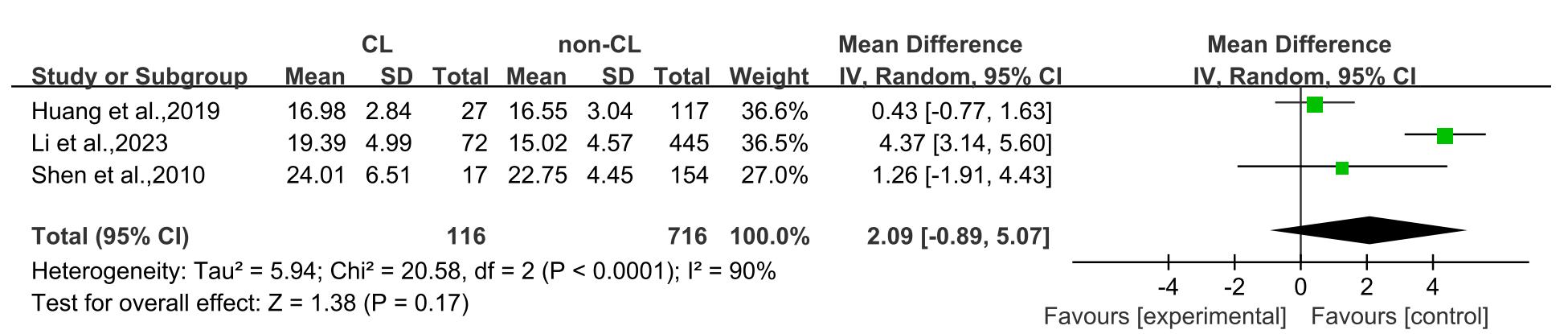


Compressed vertebral body height:


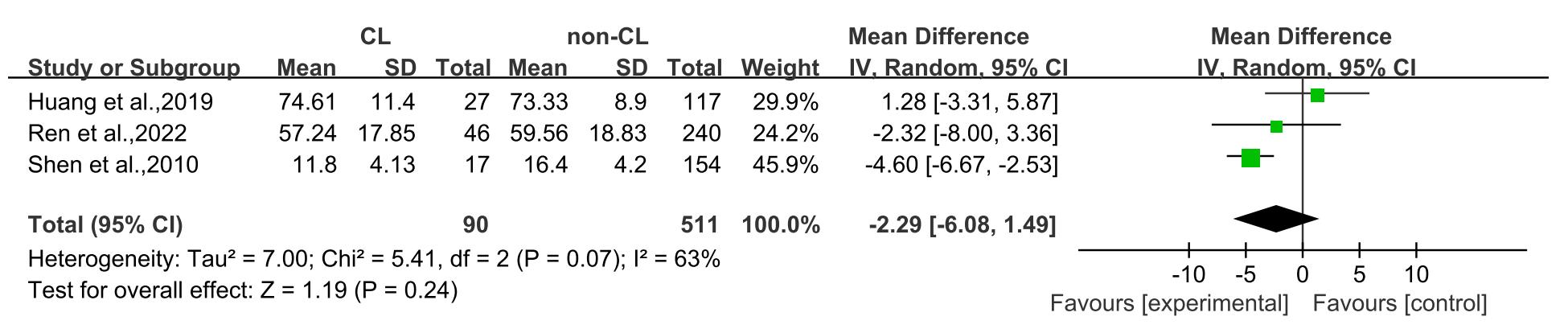


Fracture duration:


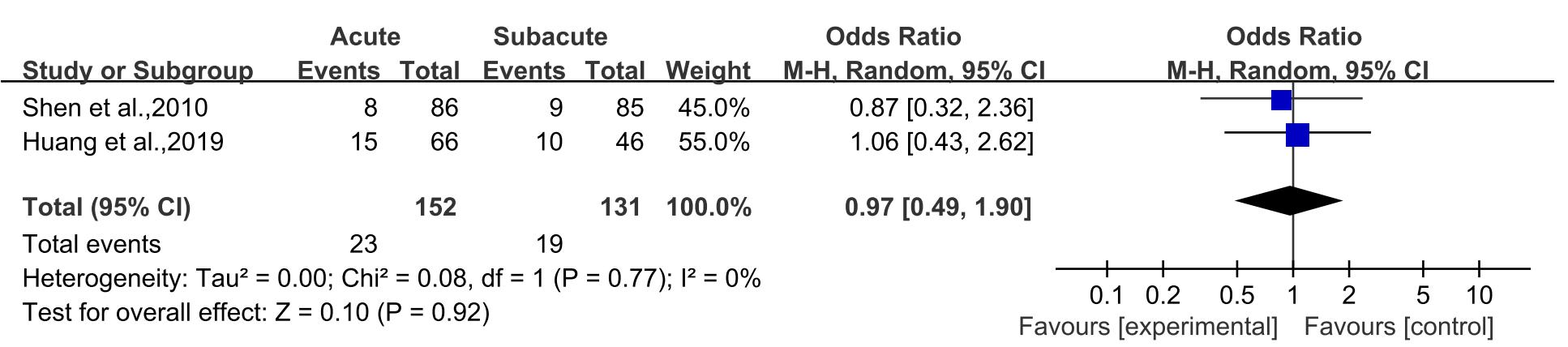


Fracture segment:


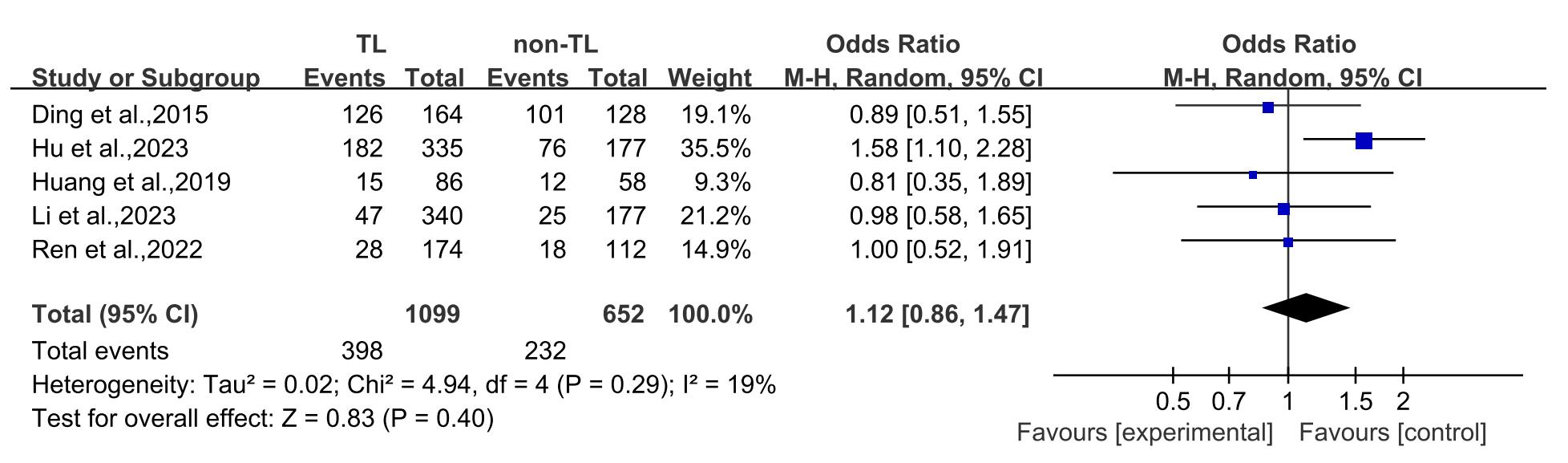


Fracture severity:


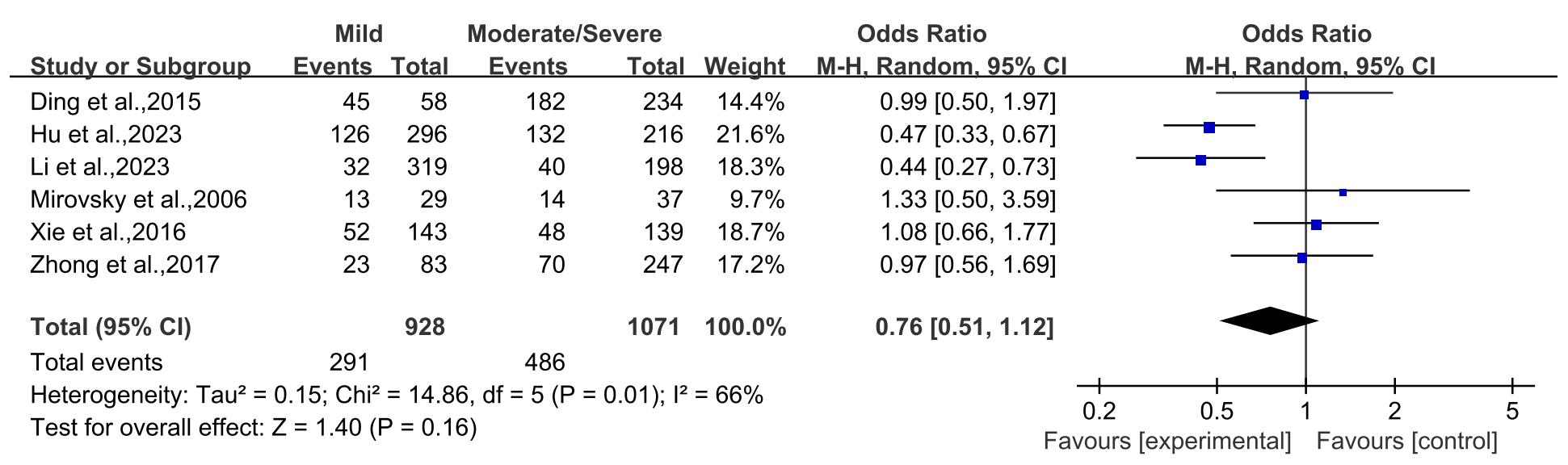


Gender:


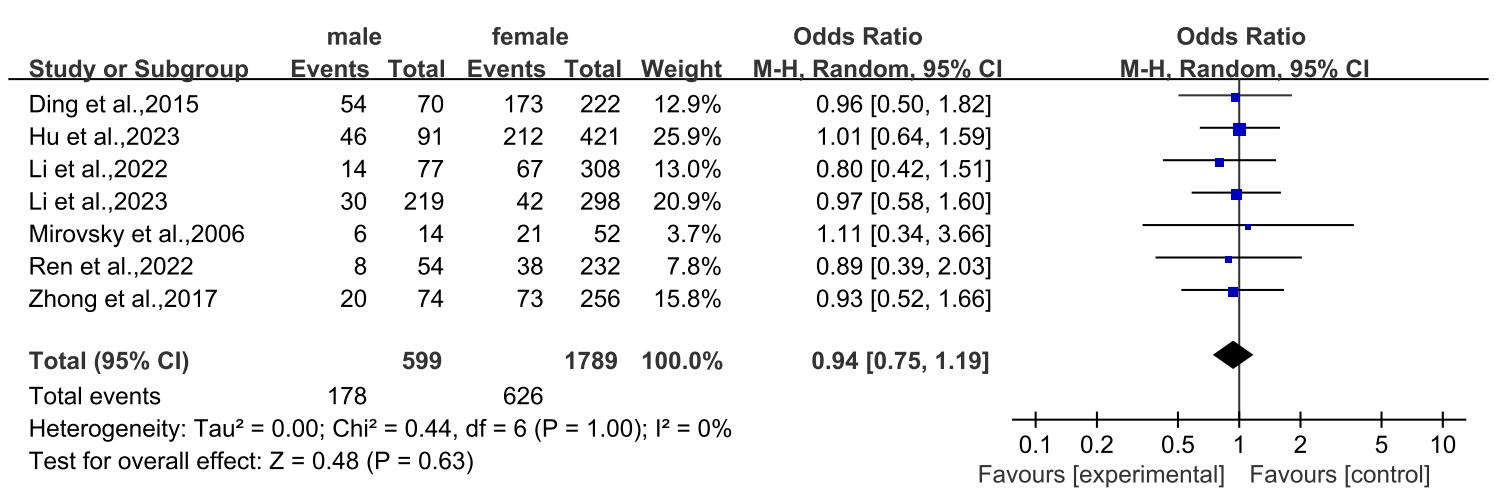


History of anti-osteoporosis treatment:


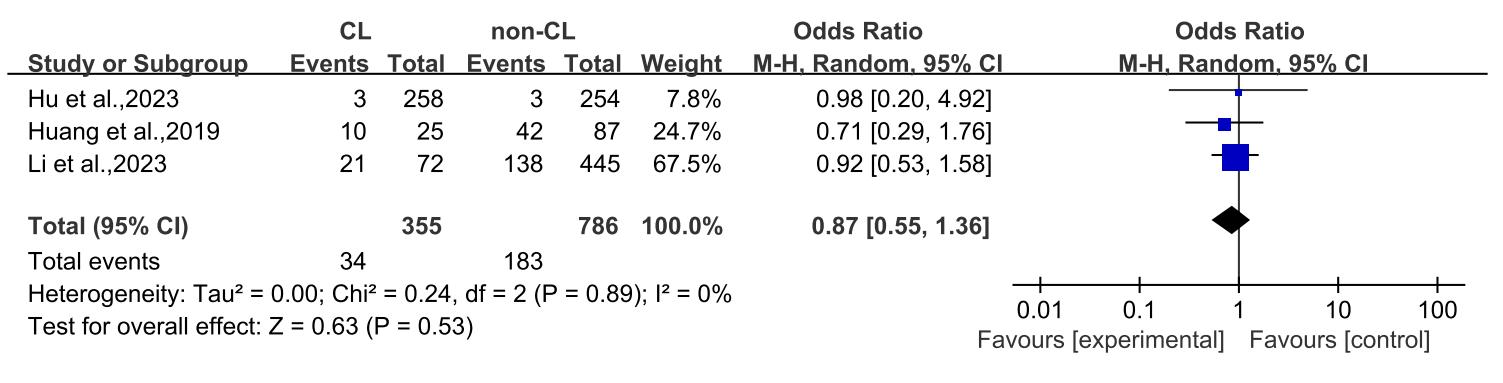


History of diabetes:


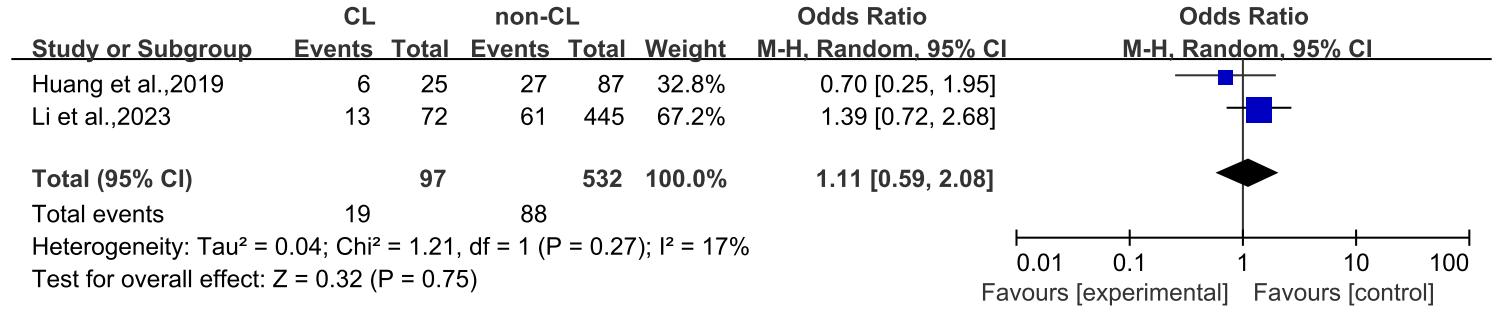


History of hypertension:


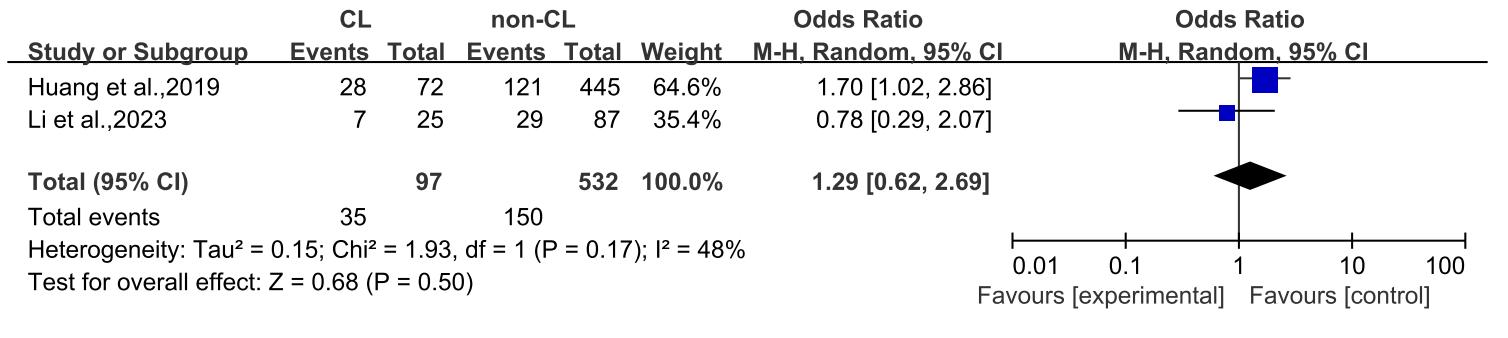


History of pedicle screw internal fixation:


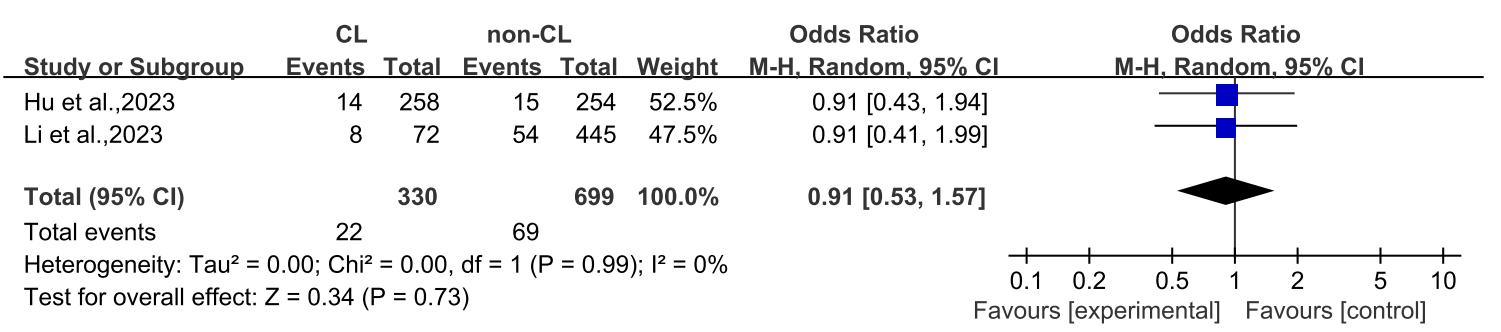


Hospitalization to surgery:


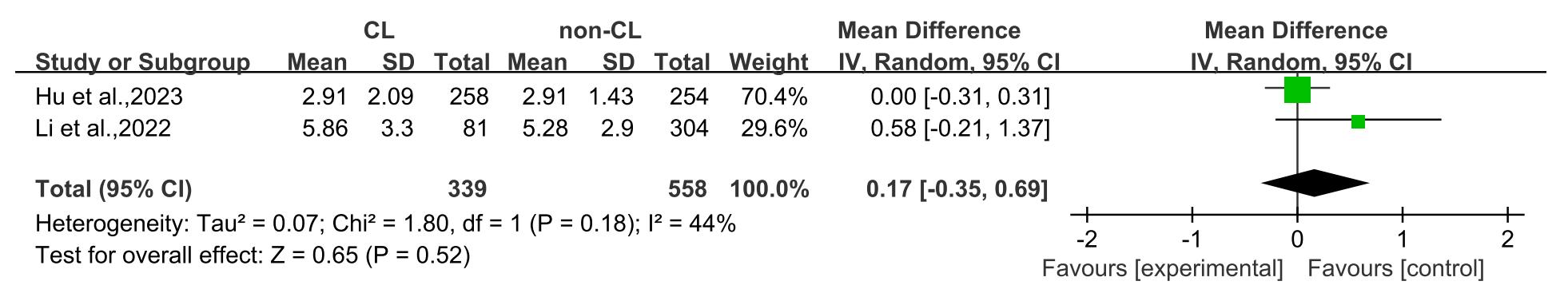


Injury to surgery:


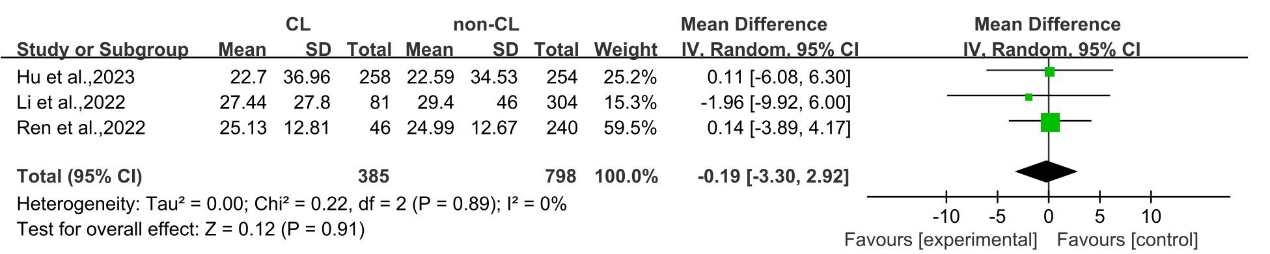


Operation time:


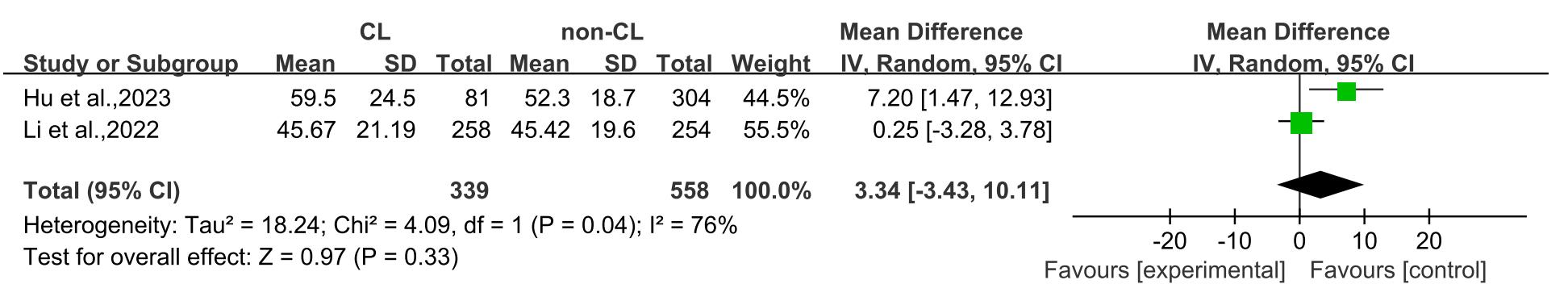


Steroid medication:


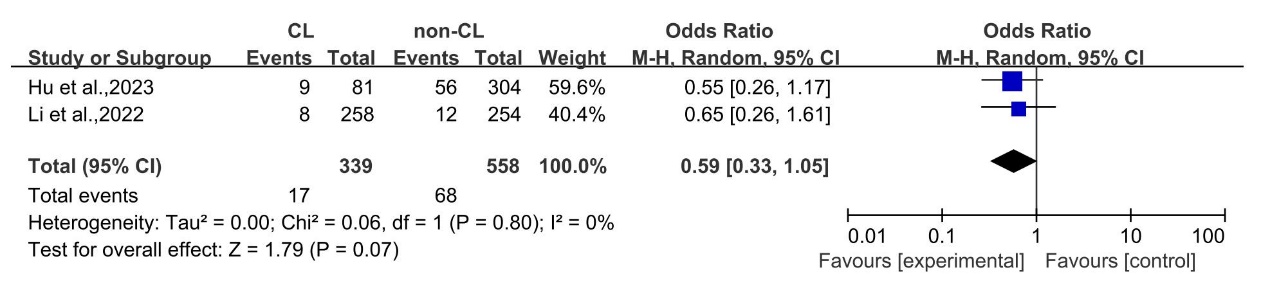


Surgical method:


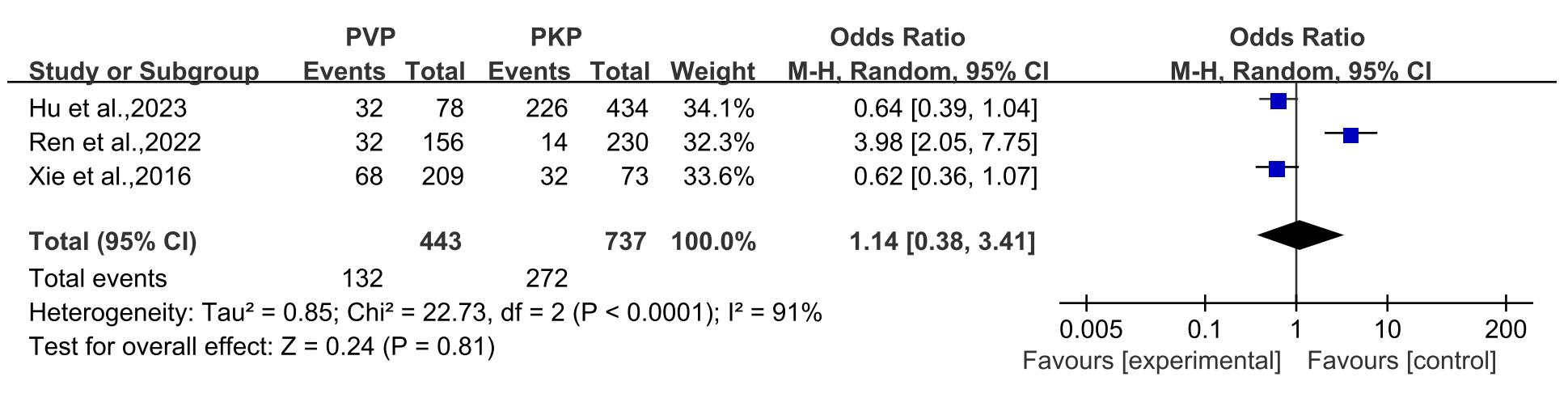

Supplement: Supplementary file 2 [file js9-111-1231-s002.docx]
